# Factors associated with unmet psychological care needs and development of a Neuman systems model-based risk prediction model in patients with hepatocellular carcinoma

**DOI:** 10.3389/fpsyt.2026.1887016

**Published:** 2026-07-10

**Authors:** Tong Wu, Qian Xu, Mugen Zhang

**Affiliations:** 1 Department of Geriatrics, The People’s Hospital of Lincang, Lincang, China; 2 Hemodialysis Unit, Ezhou Central Hospital, Ezhou, China; 3 Department of Interventional Therapy, No. 907 Hospital of the Joint Logistics Support Force, Nanping, China

**Keywords:** hepatocellular carcinoma, Neuman systems model, nomogram, oncology nursing, unmet psychological care needs

## Abstract

**Background:**

Patients with hepatocellular carcinoma (HCC) often face symptom distress, treatment decision-making, demands for family support, communication needs, and financial concerns during early hospitalization. This study examined factors associated with unmet psychological care needs and developed a risk prediction model, guided by the Neuman Systems Model, to support early nursing stratification.

**Methods:**

This single-center retrospective study included 345 adults with HCC admitted between January 2020 and December 2024. Unmet psychological care needs were defined as a score of ≥3 on any item in the psychological domain of the Chinese Supportive Care Needs Survey Short Form-34 (SCNS-SF34) within 24–48 h after admission. Candidate predictors were classified as intrapersonal, interpersonal, or extrapersonal stressors. After outcome stratification, the cohort was randomly divided into training and validation sets (7:3). Multiple imputation, least absolute shrinkage and selection operator (LASSO) selection across 20 imputed datasets, and multivariable logistic regression were used to develop the nomogram. Discrimination, calibration, decision curve analysis, bootstrap internal validation, and sensitivity analyses were evaluated.

**Results:**

Unmet psychological care needs were identified in 205 patients (59.4%). The final model retained pain numeric rating scale (NRS) score, sleep disturbance, Patient Health Questionnaire-9 (PHQ-9) score, Generalized Anxiety Disorder-7 (GAD-7) score, family support, cooperation in physician-patient communication, and perceived economic burden. Higher pain scores, sleep disturbance, higher depressive and anxiety symptom scores, poor family support, poor cooperation in physician-patient communication, and heavy perceived economic burden were independently associated with unmet psychological care needs. The areas under the receiver operating characteristic curves (AUCs) were 0.851 in the training set and 0.829 in the validation set; the bootstrap-corrected AUC was 0.836. Calibration-in-the-large, calibration slope, Brier scores, and decision curve analysis indicated acceptable apparent and internal-validation performance. Sensitivity analyses excluding PHQ-9 and GAD-7 and applying a stricter outcome definition showed consistent directions of association for the principal predictors.

**Conclusions:**

Unmet psychological care needs were common within 24–48 h after admission among patients with HCC. The internally validated nomogram may support early risk stratification, symptom management, psychosocial screening, family involvement, communication optimization, and referral to medical social work services. Independent external validation is required before routine clinical implementation.

## 1 Introduction

Hepatocellular carcinoma (HCC) remains a malignancy with a substantial global disease burden, with marked variation in incidence and mortality across geographic regions, etiologic backgrounds, and healthcare-resource settings (1). HCC commonly arises in the setting of chronic liver disease and cirrhosis, and at diagnosis patients often face multiple concurrent stressors, including tumor progression, limited hepatic functional reserve, and management of comorbidities (2).

Treatment pathways for HCC include hepatic resection, ablation, transarterial chemoembolization (TACE), systemic therapy, and combination therapy; treatment selection depends on tumor burden, liver function, performance status, and patient preferences (3). Reassessment and treatment decision-making are often concentrated early in hospitalization, requiring patients to understand prognostic information, weigh treatment risks, and arrange family caregiving and financial support within a short period (4).

In the context of oncology nursing, unmet psychological care needs refer to patients’ needs for emotional support, disease-related explanations, uncertainty management, opportunities to be heard and comforted, and coping guidance that are not adequately recognized or addressed during cancer diagnosis, treatment selection, and adaptation to hospitalization. This concept primarily emphasizes the psychological-support gap subjectively perceived by patients (5).

Unmet supportive care needs are multidimensional, and psychological needs often intersect with informational needs, symptom management, family support, and financial pressure (6). Patients with HCC may also have unmet educational, financial, transportation, emotional, and social-support needs, underscoring the need for structured screening and tiered responses during early inpatient nursing assessment (7).

In addition to tumor stage and liver function, pain, sleep disturbance, negative emotional states, family support, physician-patient communication, and perceived economic burden may influence patients’ perceptions of access to psychological care. Pain and sleep disturbance may heighten the sense of bodily threat; depressive and anxiety symptoms may indicate greater psychological vulnerability; inadequate family support and poor cooperation in physician-patient communication may undermine security and treatment understanding; and financial pressure may further amplify disease-related uncertainty.

Research on supportive care in HCC suggests that symptoms, communication, and resource arrangements should be identified early in the care pathway (8). The first 24–48 h after admission generally encompass the initial nursing assessment, explanation of the condition, treatment communication, and arrangements for caregiving and expenses. During this period, symptom, emotional, and informational needs tend to be concentrated, whereas responses to treatment and discharge outcomes have not yet occurred. Accordingly, this study prespecified this period as a clinically actionable early-inpatient screening window and used it to reduce the risk that post-outcome information would enter the prediction model.

The Neuman Systems Model conceptualizes the patient as an open system in continuous interaction with the environment and classifies stressors as intrapersonal, interpersonal, or extrapersonal, thereby accommodating disease burden, symptom experience, emotional state, family interactions, physician-patient communication, and financial pressure within one framework (9). Compared with frameworks focused on a single symptom or behavior, this model organizes multilevel stressors within a unified structure and links risk screening, targeted nursing care, and dynamic reassessment through primary, secondary, and tertiary prevention. It is therefore highly congruent with the prediction objective and nursing pathway of this study.

Current evidence on psychological care needs among patients with HCC remains predominantly descriptive, and early-inpatient risk-stratification tools that integrate a theoretical framework, routinely available nursing variables, and predictive analysis are lacking. Against this background, the present study used the Neuman Systems Model to analyze factors associated with unmet psychological care needs in patients with HCC and to develop a nomogram-based prediction model to support early psychosocial screening and individualized allocation of nursing resources (10).

## 2 Materials and methods

### 2.1 Study design and participants

This single-center retrospective study included patients with HCC who were hospitalized at our institution between January 1, 2020, and December 31, 2024. HCC was diagnosed according to relevant standards for the diagnosis and treatment of primary liver cancer and confirmed by imaging, pathology, or comprehensive clinical judgment (11). Only patients who completed an early-inpatient assessment of psychological care needs and had a clearly documented treatment plan for the index hospitalization were included. For patients with repeated admissions during the study period, only the first hospitalization with a complete assessment record was retained.

The inclusion criteria were as follows: (1) age ≥18 years; (2) HCC confirmed by imaging, pathology, or comprehensive clinical judgment; (3) completion of the initial oncology nursing assessment during the index hospitalization; (4) completion of the assessment of psychological care needs and routine psychological screening within 24–48 h after admission; and (5) clinical records sufficient to extract demographic characteristics, disease characteristics, treatment-related information, and the principal psychological-assessment measures.

The exclusion criteria were as follows: (1) intrahepatic cholangiocarcinoma, combined hepatocellular-cholangiocarcinoma, hepatic metastases, or another primary malignancy; (2) evident disturbance of consciousness, hepatic encephalopathy, severe cognitive impairment, or a language-communication disorder that precluded psychological assessment; (3) a previously established diagnosis of a severe mental disorder; (4) repeated hospitalization during the study period, with only the first admission containing a complete assessment retained; (5) missing primary-outcome data; or (6) extensive missingness in candidate predictors that could not be reliably imputed after evaluation. After screening, 345 patients were included. The study was approved by the Medical Ethics Committee of The People’s Hospital of Lincang (approval no. 2024-018). Because this was a retrospective analysis of existing medical records, the requirement for informed consent was waived. All data were de-identified before analysis, and the study was conducted in accordance with the principles of the Declaration of Helsinki.

### 2.2 Theoretical framework and definition of the primary outcome

The Neuman Systems Model conceptualizes the individual as an open system that continuously interacts with the environment, emphasizes the effects of stressors on system stability, and classifies stressors as intrapersonal, interpersonal, or extrapersonal. Nursing interventions correspond to primary, secondary, and tertiary prevention. On the basis of this framework, we established a candidate-variable structure and provisionally assigned factors that might influence psychological care needs in patients with HCC to these three stressor categories to improve the theoretical coherence of variable selection and model interpretability. Specifically, demographic characteristics, disease burden, symptom experience, and coexisting negative emotional states were categorized as intrapersonal stressors; family caregiving and social-interaction factors as interpersonal stressors; and socioeconomic and healthcare-access factors as extrapersonal stressors.

The primary outcome was the presence or absence of unmet psychological care needs. This outcome reflected patients’ subjective perception of unmet needs for psychological support, emotional reassurance, management of disease-related concerns, and help coping with uncertainty during early hospitalization. The psychological domain of the Chinese Supportive Care Needs Survey Short Form-34 (SCNS-SF34) was used to determine the outcome. The SCNS-SF34 and its Chinese version have demonstrated reliability, validity, and interpretability for assessing supportive care needs among patients with cancer (12). The SCNS-SF34 uses a 5-point scale: 1 indicates no need/not applicable, 2 indicates that the need has been met, and 3–5 indicate low, moderate, and high levels of unmet need, respectively. Accordingly, a score of ≥3 on any item in the psychological domain defined the presence of unmet psychological care needs. To evaluate the robustness of this definition, a stricter outcome was prespecified as “scores of ≥3 on at least two items in the psychological domain, or a score of ≥4 on any such item” and was used in a sensitivity analysis (13).

The first assessment of the SCNS-SF34 psychological domain completed within 24–48 h after admission was used to determine the outcome. This period corresponds to the initial nursing assessment, treatment communication, and arrangement of supportive resources, while responses to treatment, severe complications, and discharge outcomes have generally not yet occurred, thereby reducing the risk of information leakage into the prediction model. The SCNS-SF34 was administered by uniformly trained nurses using a standardized interview procedure. If a patient had difficulty writing, the nurse asked each item orally and recorded the response according to the patient’s intended meaning. If any item in the psychological domain was missing, the corresponding questionnaire assessment was considered to have a missing outcome and was excluded from the analysis.

### 2.3 Data collection and study variables

Two researchers who had received standardized training independently collected data using a prespecified extraction form; disagreements were resolved by review by a third senior nurse. All candidate variables were restricted to information available before or at the same time as outcome assessment. To prevent information leakage and model bias, treatment-outcome variables that became available only after outcome assessment were not included. For subjectively graded variables, including family support, cooperation in physician-patient communication, perceived economic burden, and transportation convenience, a standardized extraction manual was used and inter-rater agreement was calculated. Depressive symptoms were assessed with the Patient Health Questionnaire-9 (PHQ-9), which has demonstrated good structural validity and reliability for psychological screening in populations with cancer (14). Anxiety symptoms were assessed with the Generalized Anxiety Disorder-7 (GAD-7), which has acceptable psychometric properties in patients with advanced cancer (15). Recommendations for managing anxiety and depression in adults with cancer support systematic psychological screening within oncology care pathways (16).

According to the Neuman Systems Model, study variables were divided into three categories. Intrapersonal stressors included age, sex, body mass index, etiology of liver disease, cirrhosis, Charlson Comorbidity Index, Eastern Cooperative Oncology Group performance status (ECOG PS), Child-Pugh class, Barcelona Clinic Liver Cancer (BCLC) stage, alpha-fetoprotein level, maximum tumor diameter, number of tumors, portal vein tumor thrombus, ascites, pain numeric rating scale (NRS) score, sleep disturbance, fatigue, decreased appetite, and history of prior antitumor treatment. Sleep disturbance, fatigue, and decreased appetite were dichotomized according to whether their presence was explicitly documented in the initial nursing assessment or in progress notes within 24 h before or after outcome assessment. Because outcome assessment occurred within 24–48 h after admission, the treatment-related variable for the current hospitalization was recorded as the primary diagnostic or therapeutic plan already formulated or initiated at admission and was categorized as hepatic resection, ablation, transarterial chemoembolization (TACE), systemic therapy, or combination therapy.

Interpersonal stressors included marital status, living with family members, availability of a primary caregiver, the caregiver’s relationship to the patient, presence of a regular accompanying caregiver before outcome assessment, family support level, and cooperation in physician-patient communication. Family support level was categorized as good, moderate, or poor on the basis of integrated documentation in the initial nursing assessment regarding emotional support from family, participation in caregiving, and practical assistance. Cooperation in physician-patient communication was categorized as good, fair, or poor according to the initial nursing assessment and nursing handover records.

Extrapersonal stressors included educational attainment, employment status, place of residence, healthcare payment method, monthly household income, perceived economic burden, convenience of transportation to and from the hospital, and long-term healthcare outside the home region. Perceived economic burden was categorized as light, moderate, or heavy according to the initial nursing assessment. Transportation convenience was categorized as convenient, moderately convenient, or inconvenient on the basis of travel time from the patient’s residence to the hospital, transfer complexity, and nursing documentation. Because BCLC stage already integrates tumor burden and liver-function status, and portal vein tumor thrombus, maximum tumor diameter, tumor number, and Child-Pugh class are strongly clinically related to it, collinearity diagnostics and clinical interpretability were considered jointly during modeling to avoid including highly overlapping information in the final model. Categorical variables with more than two levels were dummy-coded before modeling.

### 2.4 Statistical analysis

Statistical analyses were performed using the Statistical Package for the Social Sciences (SPSS), version 26.0 (IBM Corp., Armonk, NY, USA), and R, version 4.3.0. Continuous variables were first assessed for normality. Normally distributed variables were summarized as mean ± standard deviation and compared between groups using the independent-samples *t* test; non-normally distributed variables were summarized as median *M* (*P*_25_, *P*_75_) and compared using the Mann-Whitney *U* test. Categorical variables were summarized as number (%). Categorical variables with more than two levels were compared using the Pearson *χ²* test; the Yates continuity-corrected *χ²* test was used for 2 × 2 contingency tables, and Fisher’s exact test was used when expected-frequency requirements for the *χ²* test were not met. After stratification by outcome, the sample was randomly divided into training and validation sets at a 7:3 ratio. Descriptive comparisons were based on available-case analysis. Missing data relevant to modeling were handled separately after division into the training and validation sets. Continuous variables with <5% missingness were imputed using the median, categorical variables with <5% missingness were imputed using the mode, and variables with 5%–20% missingness underwent multiple imputation by chained equations. Twenty imputed datasets were generated, with 30 iterations per dataset. Predictive mean matching was used for continuous variables, logistic regression for binary variables, and multinomial logistic regression for categorical variables with more than two levels. Convergence and plausibility were evaluated using trace plots, overlap between the distributions of imputed and observed values, the range of extreme values, and consistency with missing-data patterns. Parameter estimates were pooled according to Rubin’s rules.

Within the training set, 10-fold cross-validated least absolute shrinkage and selection operator (LASSO) regression was performed separately in each imputed dataset, and the penalty parameter was selected using the 1-standard-error criterion (lambda.1se). The prespecified variable-selection frequency threshold was ≥70%; a variable entered the candidate multivariable logistic regression model only if it was selected by LASSO in ≥70% of the imputed datasets. Selection frequencies for categorical variables with more than two levels were calculated at the overall-variable level. Candidate-model parameter estimates were pooled according to Rubin’s rules, and the final model was determined by considering clinical interpretability and parsimony. The Box-Tidwell test was used to assess linearity of continuous variables with the logit, and the variance inflation factor (VIF) was used to assess collinearity. Model performance was evaluated using the receiver operating characteristic (ROC) curve, area under the receiver operating characteristic curve (AUC), Youden index, sensitivity, specificity, Hosmer-Lemeshow test, Brier score, calibration-in-the-large, calibration slope, calibration curve, and decision curve analysis (DCA). Internal validation was performed using 1,000 bootstrap resamples. This retrospective analysis used existing cases and therefore did not include an *a priori* sample-size calculation. Events per variable (EPV) were additionally reported to describe model complexity, but EPV was not treated as the sole criterion for sample-size adequacy. The final training-set model contained 10 predictor parameters, and the candidate model contained 12. Two-sided *P* values < 0.05 were considered statistically significant.

## 3 Results

### 3.1 Comparison of baseline characteristics by outcome in the overall sample

During the study period, 512 HCC-related hospitalization records were initially retrieved. On the basis of the prespecified criteria, 74 records were excluded because the assessment of psychological care needs had not been completed within 24–48 h after admission, 21 because of repeated hospitalization during the study period, 18 because of non-HCC disease or another primary malignancy, 14 because of impaired consciousness, cognition, or language communication, 8 because of a previously diagnosed severe mental disorder, 20 because the primary outcome was missing, and 12 because candidate predictors had extensive missingness that could not be reliably imputed after evaluation. The final sample comprised 345 patients, of whom 205 had unmet psychological care needs, corresponding to a prevalence of 59.4%.

Comparison of intrapersonal stressors showed that the group with unmet psychological care needs tended to have a greater comorbidity burden, more impaired performance status, worse Child-Pugh class, more advanced BCLC stage, higher alpha-fetoprotein levels, larger maximum tumor diameter, more tumors, a higher prevalence of portal vein tumor thrombus and ascites, higher pain NRS scores, higher proportions of patients with sleep disturbance, fatigue, decreased appetite, and a history of prior antitumor treatment, a different distribution of primary treatment plans during the current hospitalization, and higher PHQ-9 and GAD-7 scores (all *P* < 0.05). Age, sex, body mass index, etiology of liver disease, and cirrhosis did not differ significantly between groups (all *P* > 0.05) (**Table 1**).

**Table 1:** Comparison of intrapersonal stressors.

Variable	Level	Unmet psychological care needs present (*n* = 205)	No unmet psychological care needs (*n* = 140)	Test statistic	*P* value
Age (years)		58.48 ± 9.10	56.89 ± 10.58	*t* = 1.450	0.148
Sex	Male	177 (86.3)	118 (84.3)	*χ²* = 0.142	0.706
	Female	28 (13.7)	22 (15.7)		
Body mass index (kg/*m*²)		22.82 ± 3.19	23.45 ± 2.80	*t* = -1.939	0.053
Etiology of liver disease	Viral hepatitis	123 (60.0)	72 (51.4)	*χ²* = 3.421	0.331
	Alcohol-related	11 (5.4)	6 (4.3)		
	Metabolic	25 (12.2)	20 (14.3)		
	Other	46 (22.4)	42 (30.0)		
Cirrhosis	Yes	124 (60.5)	79 (56.4)	*χ²* = 0.411	0.522
	No	81 (39.5)	61 (43.6)		
Charlson Comorbidity Index		2.00 (1.00, 3.00)	1.00 (1.00, 2.25)	*Z* = 3.276	<0.001
ECOG PS	0	38 (18.5)	52 (37.1)	*χ²* = 15.760	<0.001
	1	122 (59.5)	69 (49.3)		
	≥2	45 (22.0)	19 (13.6)		
Child-Pugh class	A	120 (58.5)	111 (79.3)	*χ²* = 17.815	<0.001
	B	74 (36.1)	28 (20.0)		
	C	11 (5.4)	1 (0.7)		
BCLC stage	0/A	41 (20.0)	50 (35.7)	*χ²* = 15.068	0.002
	B	79 (38.5)	56 (40.0)		
	C	70 (34.1)	27 (19.3)		
	D	15 (7.3)	7 (5.0)		
Alpha-fetoprotein (ng/mL) (*n* = 195/133)		134.82 (66.90, 295.74)	84.67 (41.82, 154.91)	*Z* = 4.362	<0.001
Maximum tumor diameter (cm)		4.91 (3.55, 6.47)	3.71 (2.75, 4.78)	*Z* = 5.158	<0.001
Number of tumors	1	86 (42.0)	84 (60.0)	*χ²* = 11.722	0.003
	2	76 (37.1)	40 (28.6)		
	≥3	43 (21.0)	16 (11.4)		
Portal vein tumor thrombus	Yes	54 (26.3)	21 (15.0)	*χ²* = 5.641	0.018
	No	151 (73.7)	119 (85.0)		
Ascites	Yes	37 (18.0)	10 (7.1)	*χ²* = 7.507	0.006
	No	168 (82.0)	130 (92.9)		
Pain NRS score (*n* = 202/138)		3.00 (2.00, 4.00)	2.00 (1.00, 3.00)	*Z* = 4.521	<0.001
Sleep disturbance	Yes	113 (55.1)	36 (25.7)	*χ²* = 28.135	<0.001
	No	92 (44.9)	104 (74.3)		
Fatigue	Yes	116 (56.6)	54 (38.6)	*χ²* = 10.092	0.001
	No	89 (43.4)	86 (61.4)		
Decreased appetite	Yes	110 (53.7)	35 (25.0)	*χ²* = 26.878	<0.001
	No	95 (46.3)	105 (75.0)		
History of prior antitumor treatment	Yes	61 (29.8)	20 (14.3)	*χ²* = 10.238	0.001
	No	144 (70.2)	120 (85.7)		
Primary treatment plan during the current hospitalization	Hepatic resection	33 (16.1)	48 (34.3)	*χ²* = 21.648	<0.001
	Ablation	17 (8.3)	17 (12.1)		
	TACE	71 (34.6)	34 (24.3)		
	Systemic therapy	44 (21.5)	28 (20.0)		
	Combination therapy	40 (19.5)	13 (9.3)		
PHQ-9 score (*n* = 201/137)		8.00 (7.00, 10.00)	4.00 (2.00, 5.00)	*Z* = 13.214	<0.001
GAD-7 score (*n* = 201/138)		6.00 (5.00, 8.00)	3.00 (2.00, 4.00)	*Z* = 11.884	<0.001

Normally distributed continuous variables are presented as mean ± standard deviation and were compared using the independent-samples *t* test, with *t* denoting the test statistic. Non-normally distributed continuous variables are presented as median *M* (*P_25_*, *P_75_*), where *P_25_* and *P_75_* denote the 25th and 75th percentiles, respectively, and were compared using the Mann-Whitney *U* test; *Z* denotes the standardized test statistic. Categorical variables are presented as number (%). Categorical variables with more than two levels were compared using the Pearson *χ²* test; the Yates continuity-corrected *χ²* test was used for 2 × 2 contingency tables, and Fisher’s exact test was used when expected-frequency requirements for the *χ²* test were not met; *χ²* denotes the chi-square statistic. The *n* following a variable name indicates the nonmissing sample size in the corresponding group, and percentages use nonmissing cases as the denominator. All *P* values are two-sided. ECOG PS, Eastern Cooperative Oncology Group performance status; BCLC, Barcelona Clinic Liver Cancer; NRS, numeric rating scale; TACE, transarterial chemoembolization; PHQ-9, Patient Health Questionnaire-9; GAD-7, Generalized Anxiety Disorder-7.

Comparison of interpersonal stressors showed that the group with unmet psychological care needs had lower proportions of patients living with family members and having a regular accompanying caregiver, as well as poorer family support and cooperation in physician-patient communication (all *P* < 0.05). Marital status, availability of a primary caregiver, and the relationship of the primary caregiver to the patient did not differ significantly between groups (all *P* > 0.05) (**Table 2**).

**Table 2:** Comparison of interpersonal stressors.

Variable	Level	Unmet psychological care needs present (*n* = 205)	No unmet psychological care needs (*n* = 140)	Test statistic	*P* value
Marital status	Married/cohabiting	163 (79.5)	111 (79.3)	*χ²* = 0.000	1.000
	Other	42 (20.5)	29 (20.7)		
Living with family members	Yes	153 (74.6)	118 (84.3)	*χ²* = 4.044	0.044
	No	52 (25.4)	22 (15.7)		
Primary caregiver available	Yes	185 (90.2)	134 (95.7)	*χ²* = 2.831	0.092
	No	20 (9.8)	6 (4.3)		
Relationship of primary caregiver	Spouse	117 (57.1)	83 (59.3)	*χ²* = 3.978	0.264
	Children	50 (24.4)	35 (25.0)		
	Other	18 (8.8)	16 (11.4)		
	No primary caregiver	20 (9.8)	6 (4.3)		
Regular accompanying caregiver	Yes	120 (58.5)	115 (82.1)	*χ²* = 20.272	<0.001
	No	85 (41.5)	25 (17.9)		
Family support level (*n* = 193/132)	Good	51 (26.4)	77 (58.3)	*χ²* = 36.043	<0.001
	Moderate	84 (43.5)	40 (30.3)		
	Poor	58 (30.1)	15 (11.4)		
Cooperation in physician-patient communication (*n* = 195/132)	Good	51 (26.2)	78 (59.1)	*χ²* = 39.281	<0.001
	Fair	102 (52.3)	46 (34.8)		
	Poor	42 (21.5)	8 (6.1)		

Categorical variables are presented as number (%). Categorical variables with more than two levels were compared using the Pearson *χ²* test; the Yates continuity-corrected *χ²* test was used for 2 × 2 contingency tables, and Fisher’s exact test was used when expected-frequency requirements for the *χ²* test were not met; *χ²* denotes the chi-square statistic. The *n* following a variable name indicates the nonmissing sample size in the corresponding group, and percentages use nonmissing cases as the denominator. All *P* values are two-sided. Family support level and cooperation in physician-patient communication were both three-level ordinal variables.

Comparison of extrapersonal stressors showed less favorable distributions of educational attainment, employment status, healthcare payment method, monthly household income, perceived economic burden, and transportation convenience in the group with unmet psychological care needs (all *P* < 0.05). Place of residence and long-term healthcare outside the home region did not differ significantly between groups (all *P* > 0.05) (**Table 3**).

**Table 3:** Comparison of extrapersonal stressors.

Variable	Level	Unmet psychological care needs present (*n* = 205)	No unmet psychological care needs (*n* = 140)	Test statistic	*P* value
Educational attainment	Middle school or below	82 (40.0)	32 (22.9)	*χ²* = 13.443	0.001
	High school/technical secondary school	76 (37.1)	56 (40.0)		
	College or above	47 (22.9)	52 (37.1)		
Employment status (*n* = 203/138)	Employed	59 (29.1)	38 (27.5)	*χ²* = 7.644	0.022
	Retired	73 (36.0)	33 (23.9)		
	Unemployed	71 (35.0)	67 (48.6)		
Place of residence	Urban	80 (39.0)	70 (50.0)	*χ²* = 3.643	0.056
	Rural	125 (61.0)	70 (50.0)		
Healthcare payment method	Medical insurance	144 (70.2)	112 (80.0)	*χ²* = 7.279	0.026
	Self-pay	33 (16.1)	21 (15.0)		
	Other	28 (13.7)	7 (5.0)		
Monthly household income (*n* = 189/129)	<5000 CNY	62 (32.8)	23 (17.8)	*χ²* = 17.436	<0.001
	5000–9999 CNY	85 (45.0)	51 (39.5)		
	≥10000 CNY	42 (22.2)	55 (42.6)		
Perceived economic burden (*n* = 198/135)	Light	40 (20.2)	46 (34.1)	*χ²* = 8.998	0.011
	Moderate	82 (41.4)	52 (38.5)		
	Heavy	76 (38.4)	37 (27.4)		
Convenience of transportation to and from the hospital (*n* = 192/131)	Convenient	59 (30.7)	64 (48.9)	*χ²* = 19.149	<0.001
	Moderately convenient	70 (36.5)	50 (38.2)		
	Inconvenient	63 (32.8)	17 (13.0)		
Long-term healthcare outside the home region	Yes	59 (28.8)	32 (22.9)	*χ²* = 1.213	0.271
	No	146 (71.2)	108 (77.1)		

Categorical variables are presented as number (%). Categorical variables with more than two levels were compared using the Pearson *χ²* test; the Yates continuity-corrected *χ²* test was used for 2 × 2 contingency tables, and Fisher’s exact test was used when expected-frequency requirements for the *χ²* test were not met; *χ²* denotes the chi-square statistic. The *n* following a variable name indicates the nonmissing sample size in the corresponding group, and percentages use nonmissing cases as the denominator. All *P* values are two-sided. CNY, Chinese yuan. Perceived economic burden and convenience of transportation to and from the hospital were both three-level ordinal variables.

### 3.2 Training and validation sets, missing data, and agreement for subjective variables

The training and validation sets did not differ significantly in outcome distribution, age, sex, BCLC stage, primary treatment plan at admission, PHQ-9 score, GAD-7 score, family support level, or perceived economic burden (all *P* > 0.05) (**Table 4**). Missingness in candidate variables was low and predominantly sporadic; the primary outcome was complete, and no modeling variable had >20% missingness (**Supplementary Table 1**). Inter-rater agreement for the subjectively graded variables was good (**Supplementary Table 2**).

**Table 4:** Comparison of key characteristics between the training and validation sets.

Variable	Level	Training set (*n* = 241)	Validation set (*n* = 104)	Test statistic	*P* value
Outcome	No unmet psychological care needs	98 (40.7)	42 (40.4)	*χ²* = 0.000	1.000
	Unmet psychological care needs present	143 (59.3)	62 (59.6)		
Age (years)		57.42 ± 9.71	58.78 ± 9.81	*t* = -1.182	0.239
Sex	Male	205 (85.1)	90 (86.5)	*χ²* = 0.036	0.849
	Female	36 (14.9)	14 (13.5)		
BCLC stage	0/A	64 (26.6)	27 (26.0)	*χ²* = 0.472	0.925
	B	92 (38.2)	43 (41.3)		
	C	70 (29.0)	27 (26.0)		
	D	15 (6.2)	7 (6.7)		
Primary treatment plan during the current hospitalization	Hepatic resection	53 (22.0)	28 (26.9)	*χ²* = 3.383	0.496
	Ablation	21 (8.7)	13 (12.5)		
	TACE	75 (31.1)	30 (28.8)		
	Systemic therapy	51 (21.2)	21 (20.2)		
	Combination therapy	41 (17.0)	12 (11.5)		
PHQ-9 score (*n* = 236/102)		7.00 (4.00, 9.00)	6.00 (4.00, 9.00)	*Z* = 0.287	0.774
GAD-7 score (*n* = 237/102)		5.00 (3.00, 7.00)	5.00 (3.00, 8.00)	*Z* = 0.851	0.395
Family support level (*n* = 227/98)	Good	86 (37.9)	42 (42.9)	*χ²* = 0.870	0.647
	Moderate	90 (39.6)	34 (34.7)		
	Poor	51 (22.5)	22 (22.4)		
Perceived economic burden (*n* = 233/100)	Light	60 (25.8)	26 (26.0)	*χ²* = 0.004	0.998
	Moderate	94 (40.3)	40 (40.0)		
	Heavy	79 (33.9)	34 (34.0)		

Normally distributed continuous variables are presented as mean ± standard deviation and were compared using the independent-samples *t* test. Non-normally distributed continuous variables are presented as median *M* (*P_25_*, *P_75_*) and were compared using the Mann-Whitney *U* test. Categorical variables are presented as number (%). Categorical variables with more than two levels were compared using the Pearson *χ²* test; the Yates continuity-corrected *χ²* test was used for 2 × 2 contingency tables, and Fisher’s exact test was used when expected-frequency requirements for the *χ²* test were not met. The *n* following a variable name indicates the nonmissing sample size in the training/validation set, and percentages use nonmissing cases in the corresponding dataset as the denominator. All *P* values are two-sided. BCLC, Barcelona Clinic Liver Cancer; TACE, transarterial chemoembolization; PHQ-9, Patient Health Questionnaire-9; GAD-7, Generalized Anxiety Disorder-7.

### 3.3 Feature selection, candidate model, and final model

Pain NRS score, PHQ-9 score, and GAD-7 score showed no evidence of departure from linearity with the logit (all *P* > 0.05) (**Supplementary Table 3**). Across the 20 imputed datasets, PHQ-9 score and sleep disturbance had the highest LASSO selection frequencies. GAD-7 score, pain NRS score, family support level, cooperation in physician-patient communication, ECOG PS, and perceived economic burden also reached the prespecified selection-frequency threshold, whereas decreased appetite, BCLC stage, fatigue, primary treatment plan at admission, ascites, portal vein tumor thrombus, and monthly household income did not (**Supplementary Table 4**).

In the candidate model containing ECOG PS, effect sizes were expressed as odds ratios (ORs) with 95% confidence intervals (CIs). Compared with ECOG PS = 0, neither ECOG PS = 1 (OR = 1.39, 95% CI: 0.70–2.79, *P* = 0.350) nor ECOG PS ≥2 (OR = 1.59, 95% CI: 0.66–3.80, *P* = 0.300) reached statistical significance; the overall Wald test for ECOG PS, with 2 degrees of freedom, yielded *P* = 0.546. After ECOG PS was removed, the maximum relative change in the regression coefficients of the remaining variables was 5.6%, and the training-set AUC changed from 0.853 to 0.851. ECOG PS was therefore not included in the final nomogram (**Table 5**).

**Table 5:** ECOG PS parameters in the candidate model and the final multivariable logistic regression model.

Section/Variable	*β*	SE	OR (95% CI)	*P* value	Interpretation
A. Effect of ECOG PS in the candidate model containing ECOG PS					
ECOG PS: 1 vs 0	0.331	0.354	1.39 (0.70–2.79)	0.350	ECOG PS = 0 as the reference
ECOG PS: ≥2 vs 0	0.462	0.446	1.59 (0.66–3.80)	0.300	ECOG PS = 0 as the reference
Overall Wald test for ECOG PS	—	—	—	0.546	Overall test with 2 degrees of freedom; candidate-model training-set AUC = 0.853
B. Final model (ECOG PS not included)					
Intercept	-3.020	0.612	—	<0.001	Used in the model equation
Pain NRS score (per 1-point increase)	0.215	0.070	1.24 (1.08–1.42)	0.002	Continuous variable
Sleep disturbance (yes vs no)	0.728	0.286	2.07 (1.18–3.62)	0.011	No as the reference
PHQ-9 score (per 1-point increase)	0.148	0.038	1.16 (1.08–1.25)	<0.001	Continuous variable
GAD-7 score (per 1-point increase)	0.113	0.044	1.12 (1.03–1.22)	0.010	Continuous variable
Family support: moderate vs good	0.307	0.283	1.36 (0.78–2.36)	0.278	Good as the reference
Family support: poor vs good	0.880	0.348	2.41 (1.22–4.77)	0.011	Good as the reference
Cooperation in physician-patient communication: fair vs good	0.399	0.296	1.49 (0.84–2.66)	0.180	Good as the reference
Cooperation in physician-patient communication: poor vs good	0.928	0.398	2.53 (1.16–5.51)	0.019	Good as the reference
Perceived economic burden: moderate vs light	0.255	0.303	1.29 (0.71–2.35)	0.405	Light as the reference
Perceived economic burden: heavy vs light	0.637	0.319	1.89 (1.01–3.52)	0.046	Light as the reference

Part A reports only the ECOG PS parameters from the candidate model containing ECOG PS, whereas Part B reports the complete final model after removal of ECOG PS; the two parts should not be interpreted as one set of coefficients. *β* denotes the logistic regression coefficient; SE, standard error; OR, odds ratio; CI, confidence interval; AUC, area under the receiver operating characteristic curve. The Wald test was used to assess an individual regression coefficient or the overall effect of a categorical variable. All *P* values are two-sided. ECOG PS, Eastern Cooperative Oncology Group performance status; NRS, numeric rating scale; PHQ-9, Patient Health Questionnaire-9; GAD-7, Generalized Anxiety Disorder-7.

The final model showed that a higher pain NRS score, sleep disturbance, a higher PHQ-9 score, a higher GAD-7 score, poor family support, poor cooperation in physician-patient communication, and heavy perceived economic burden were independently associated with unmet psychological care needs (all *P* < 0.05) (**Table 5**).

**Figure 1:**
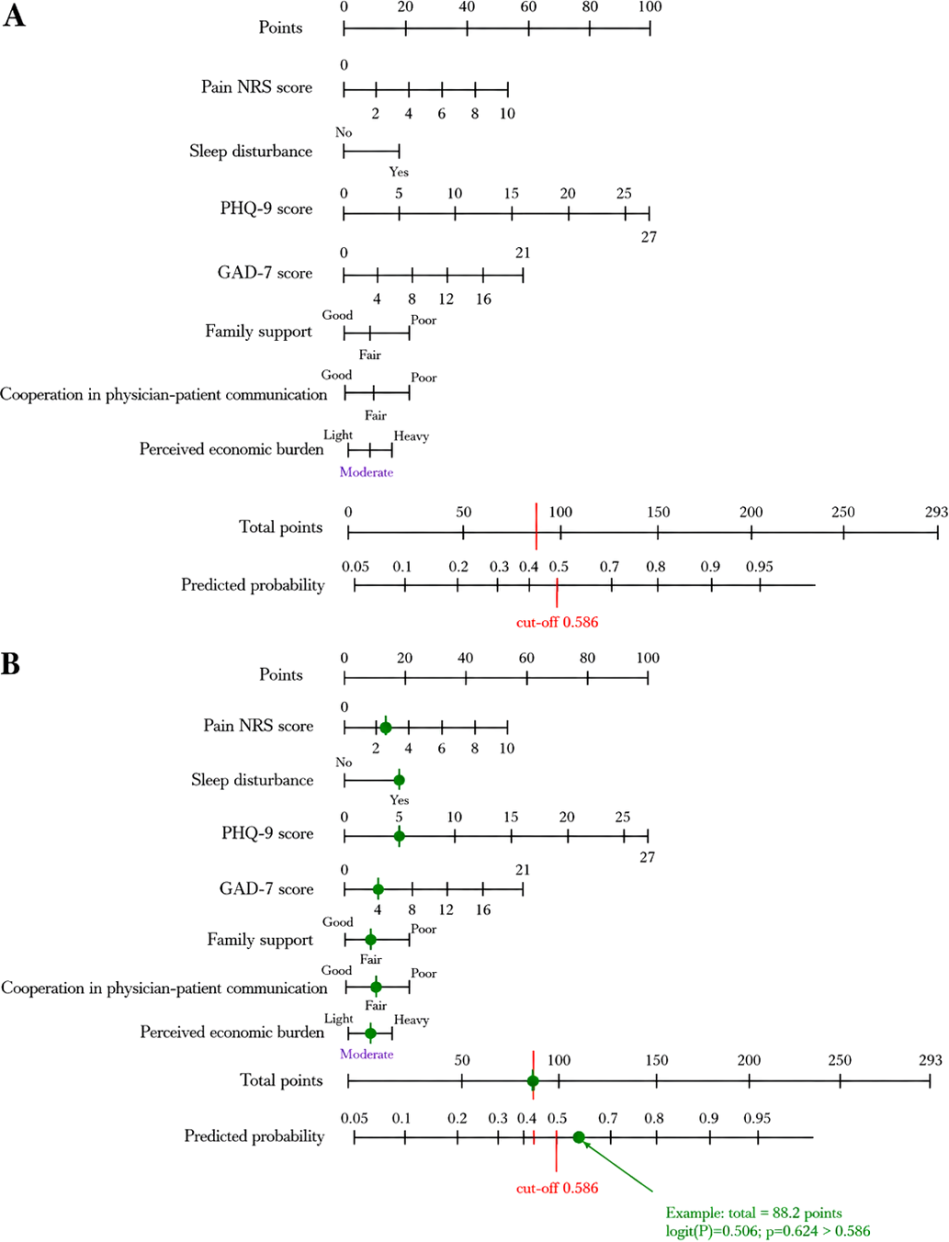
Nomogram constructed from the final multivariable logistic regression model. **(A)**, nomogram without an example overlay; **(B)**, example of risk calculation and interpretation. For the example patient assessed within 24–48 h after admission, the pain NRS score was 3, sleep disturbance was present, the PHQ-9 score was 5, the GAD-7 score was 4, family support was moderate, cooperation in physician-patient communication was fair, and perceived economic burden was moderate. Using the regression coefficients in **Table 5**B, logit(*p*) = 0.506, predicted probability *p* = 0.624, and the total nomogram score is approximately 88.2 points. Because this probability exceeds the internally derived cutoff of 0.586, the patient is classified as high risk. For binary variables, “yes” was coded as 1 and “no” as 0. Good family support, good cooperation in physician-patient communication, and light perceived economic burden were the reference categories; dummy variables not represented in the example were coded as 0. NRS, numeric rating scale; PHQ-9, Patient Health Questionnaire-9; GAD-7, Generalized Anxiety Disorder-7.

The linear predictor for the final model was: logit(*p*) = −3.020 + 0.215 × pain NRS score + 0.728 × sleep disturbance + 0.148 × PHQ-9 score + 0.113 × GAD-7 score + 0.307 × moderate family support + 0.880 × poor family support + 0.399 × fair cooperation in physician-patient communication + 0.928 × poor cooperation in physician-patient communication + 0.255 × moderate perceived economic burden + 0.637 × heavy perceived economic burden. A nomogram was constructed from this equation (**Figure 1**). **Figure 1A** shows the nomogram without an example overlay, whereas **Figure 1B** provides an annotated example of risk calculation and interpretation.

### 3.4 Model performance, calibration, decision curves, and sensitivity analyses

**Figure 2:**
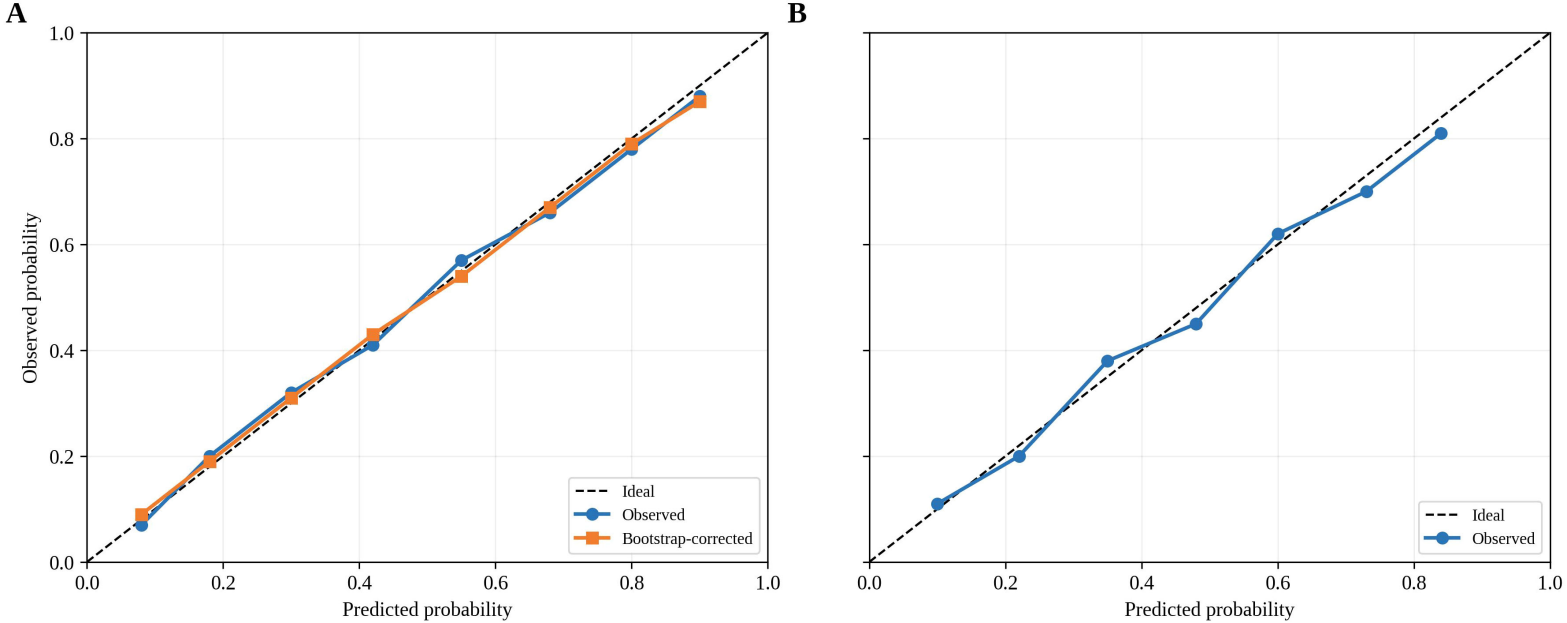
Calibration curves for the final model in the training and validation sets. **(A)**, training set; **(B)**, validation set. The x-axis represents predicted probability and the y-axis represents observed probability. Closer proximity of the curve to the 45° ideal reference line indicates better agreement between predicted and observed probabilities.

**Figure 3:**
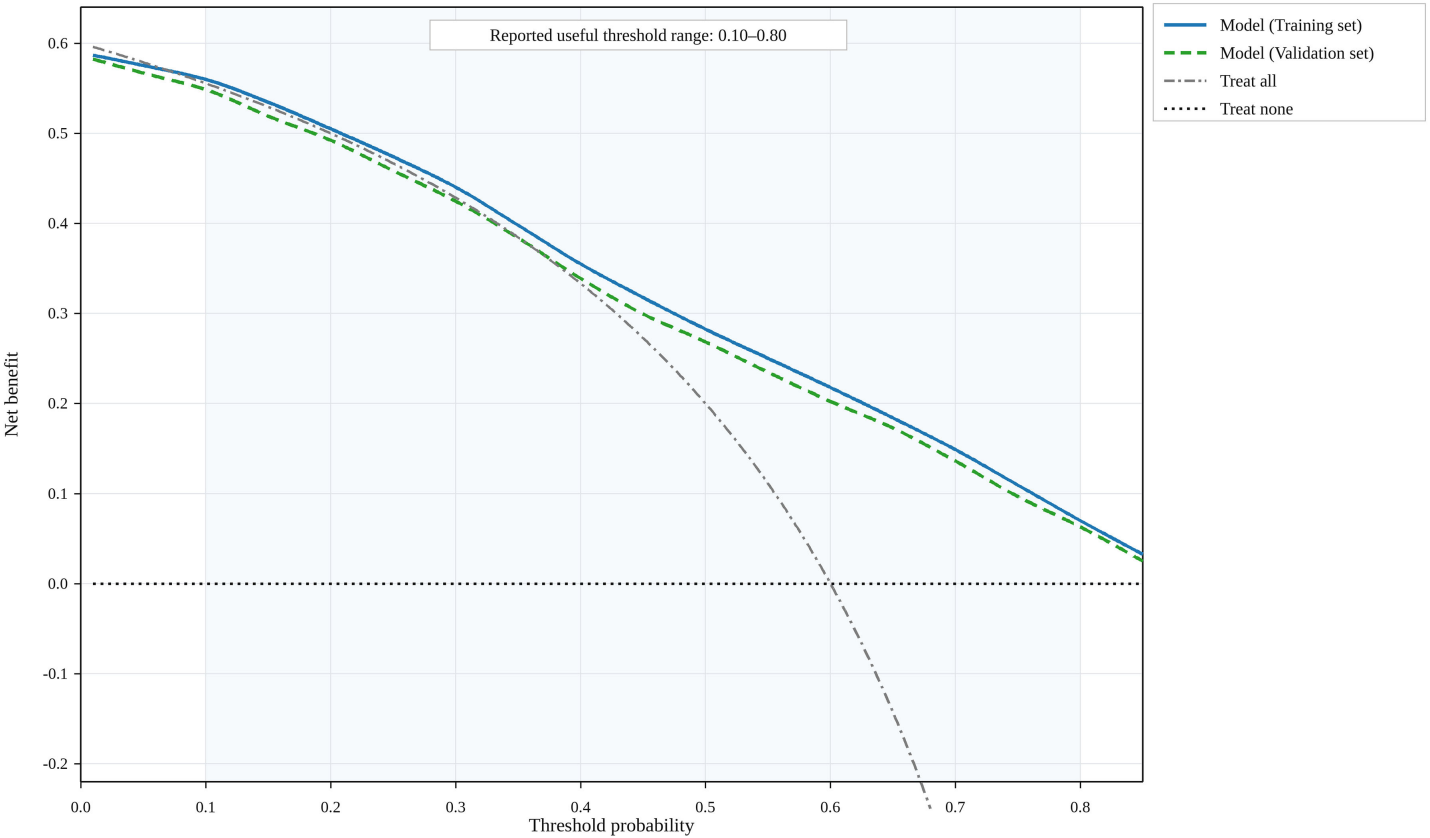
Decision curve analysis (DCA). The x-axis represents threshold probability and the y-axis represents net benefit. When the model curve lies above the treat-all and treat-none reference lines, the model provides greater net benefit within the corresponding threshold range. In both the training and validation sets, the model curves were above both reference strategies across threshold probabilities of 0.10–0.80.

The final model showed good discrimination in both the training and validation sets, and the bootstrap-corrected AUC was slightly lower than the apparent training-set AUC. The optimal cutoff in the training set was 0.586. When the same cutoff was applied to the validation set, sensitivity, specificity, and the Youden index remained acceptable. The Hosmer-Lemeshow test, Brier score, calibration-in-the-large, and calibration slope indicated acceptable calibration (**Table 6**; **Figure 2**). Decision curve analysis showed that the model curves for both the training and validation sets were above the treat-all and treat-none reference strategies across threshold probabilities of 0.10–0.80 (**Figure 3**).

**Table 6:** Model performance.

Metric	Training set	Validation set (using the training-set cutoff)	Bootstrap-corrected training set
AUC (95% CI)	0.851 (0.801–0.901)	0.829 (0.748–0.891)	0.836
Classification cutoff	0.586	0.586	—
Sensitivity (%)	78.3	74.2	—
Specificity (%)	74.5	71.4	—
Youden index	0.528	0.456	—
Hosmer-Lemeshow test *P* value	0.624	0.753	—
Brier score	0.153	0.167	—
Calibration-in-the-large	0.000 (-0.047–0.049)	0.027 (-0.096–0.150)	0.018
Calibration slope	0.962 (0.833–1.091)	0.914 (0.751–1.077)	0.936

AUC, area under the receiver operating characteristic curve; CI, confidence interval. The Hosmer-Lemeshow test assesses grouped goodness-of-fit; a large *P* value does not establish perfect fit. The Brier score is the mean squared error between predicted probabilities and observed outcomes, with lower values indicating lower overall prediction error. Calibration-in-the-large reflects systematic deviation between mean predicted risk and observed risk, with an ideal value of 0. The calibration slope reflects the scaling of predictions, with an ideal value of 1. Youden index = sensitivity + specificity − 1. The validation set used the cutoff of 0.586 determined in the training set. Bootstrap correction was based on 1,000 resamples. All *P* values are two-sided.

After PHQ-9 and GAD-7 were excluded, model discrimination decreased but remained moderate, and the directions of association for pain, sleep disturbance, family support, physician-patient communication, and perceived economic burden remained consistent (**Supplementary Table 5**). Under the stricter outcome definition, 169 patients were classified as having unmet psychological care needs, corresponding to a prevalence of 49.0%; the directions of association for the principal predictors also remained consistent (**Supplementary Table 6**).

## 4 Discussion

This study found that 59.4% of patients with HCC had unmet psychological care needs within 24–48 h after admission, indicating that gaps in psychological care are already prominent early in hospitalization. More severe negative emotional states early in admission were associated with a greater likelihood of reporting inadequately met psychological support, consistent with evidence on dynamic changes in psychiatric symptoms and quality of life during HCC treatment (17). Pain NRS score remained in the final model, suggesting that pain not only represents a physical symptom but may also intensify patients’ perceptions of disease threat and treatment uncertainty. HCC-related symptoms should therefore be actively assessed and managed early in hospitalization to prevent physical symptom burden from compounding unmet psychological care needs (18). Sleep disturbance was another important predictor. Sleep impairment in patients with HCC may reflect the combined effects of the disease itself, liver-function status, treatment-related stress, and the inpatient environment; inadequate nocturnal rest may weaken daytime coping and increase the need for emotional support and explanatory communication (19). Sleep disturbance, fatigue, and depressed mood frequently co-occur in patients with cancer and may have additive effects on symptom burden and emotional distress. These findings support concurrent assessment of sleep, pain, fatigue, and negative emotional states early in HCC hospitalization rather than treating psychological care needs in isolation (20).

Poor family support and heavy perceived economic burden were both associated with unmet psychological care needs, indicating that psychological-support gaps arise not only from the disease itself but also from caregiving resources and financial pressure. Social support and financial toxicity affect quality of life, confidence in treatment, and perceived access to care among patients with HCC (21). Poor cooperation in physician-patient communication also remained in the final model, underscoring communication quality as a key component of psychological care early in hospitalization. When patients do not adequately understand their condition, treatment objectives, potential benefits and risks, and subsequent arrangements, substantial psychological care gaps may persist even after routine care has begun (22). Unmet supportive care needs are closely related to poorer quality of life, and psychological needs commonly coexist with informational, physical, care-related, and social-support needs. Unmet psychological care needs may therefore serve as an early signal of imbalance within a multidimensional care system (23).

Patient-reported outcomes and quality-of-life assessments in HCC can help clinicians more accurately capture symptom burden, psychological distress, and treatment preferences. Moving assessment of psychological care needs to the early inpatient period can convert subjective patient experiences into actionable nursing information (24). Evidence on postoperative quality of life in HCC suggests that physical status, psychological status, socioeconomic conditions, and symptom burden may all influence subsequent quality of life. In the present study, tumor-burden variables were significant in univariable analyses but did not enter the final model, suggesting that early psychological needs more closely reflect the combined effects of symptom experience, emotional state, and available support resources (25). Quality of life among patients with HCC receiving treatments such as TACE is influenced by demographic, clinical, psychological, social, and symptom-related factors. The variables in our model similarly span these domains, supporting the use of a multidimensional stressor framework to interpret unmet psychological care needs (26). In unresectable HCC, symptom and quality-of-life measures reflect patients’ lived experiences during treatment. Including routinely available nursing variables in the prediction model facilitates risk stratification early in the treatment course and preserves time for subsequent communication and intervention (27). Evidence related to locoregional treatment and radioembolization indicates that quality of life and survival outcomes are influenced by symptoms, treatment modality, and patient status. This supports concurrent assessment of symptom control, psychological needs, and subsequent nursing-resource needs after risk stratification (28). Patients’ experiences of HCC and treatment goals frequently center on symptom control, prognostic uncertainty, and maintenance of daily functioning. Integrating variables related to these experiences within one model enhances the clinical interpretability of the nomogram (29). Data on systemic therapy show associations between adverse events and quality of life, indicating that symptom identification during treatment should proceed in parallel with assessment of the patient experience. The inclusion of pain, sleep disturbance, and negative emotional states in the final model is consistent with patient-reported outcome management (30).

Standards for survivorship care in advanced or metastatic cancer emphasize symptom management, psychosocial support, care coordination, and continuous communication. A risk-stratified nursing pathway is aligned with these patient-centered care goals (31). Prediction models in oncology nursing can integrate multiple routinely collected variables into individualized risk estimates. Compared with screening using a single scale, joint modeling of symptoms, emotions, family factors, communication, and financial pressure better supports a tiered nursing pathway (32). Reporting standards for prediction models emphasize transparent definitions of variables, handling of missing data, presentation of the complete model equation, discrimination, calibration, and clinical utility. Concurrent reporting of LASSO selection stability, the imputation process, calibration slope, calibration-in-the-large, and sensitivity analyses improves reproducibility (33). The training set contained 143 events, and the final model included 10 predictor parameters, yielding an EPV of 14.3; the candidate model containing ECOG PS included 12 predictor parameters, yielding an EPV of 11.9. EPV describes the relation between the number of events and the number of model parameters but cannot replace a formal sample-size assessment based on outcome proportion, anticipated model performance, and control of overfitting (34). Because no *a priori* sample-size calculation was performed in this retrospective study, model stability must still be tested in independent samples.

Multidisciplinary care can address the complex decision-making and supportive care needs inherent in HCC management. Risk-stratification results may facilitate coordination among the primary nurse, attending physician, psycho-oncology nursing services, medical social work, and medical insurance or financial-assistance counseling, consistent with the direction of comprehensive HCC care (35). The implementation quality of tumor boards and multidisciplinary discussions can influence adherence to HCC treatment recommendations. For patients with poor cooperation in physician-patient communication or limited treatment understanding, joint communication by the primary nurse and attending physician, teach-back confirmation, and multidisciplinary rounds when necessary can translate risk identification into care actions (36). For clinical use, the model could be embedded in the early inpatient oncology nursing assessment workflow. Within 24–48 h after admission, nurses could assess pain NRS score, sleep disturbance, PHQ-9, GAD-7, family support, cooperation in physician-patient communication, and perceived economic burden, and calculate predicted probability through the electronic medical record or nomogram. Patients with a predicted probability ≥0.586 could be classified as high risk, triggering a tiered nursing response within 24 h: reassessment of the analgesic regimen for patients with higher pain NRS scores; sleep-hygiene counseling, nighttime environmental optimization, and pharmacologic assessment when necessary for those with sleep disturbance; consultation with a psycho-oncology nurse specialist or referral for psychiatric or psychological assessment for those with higher PHQ-9 or GAD-7 scores; a family meeting and clarification of the primary caregiver’s role for those with poor family support; joint communication involving the primary nurse and attending physician, teach-back confirmation, and multidisciplinary rounds when necessary for those with poor cooperation in physician-patient communication; and referral to medical social work or counseling regarding medical insurance or financial-assistance programs for those with heavy perceived economic burden. Reassessment could be performed within 72 h when the treatment plan changes, symptoms worsen, or family caregiving circumstances change. This workflow is an implementation proposal based on internal-validation results and requires external validation and local adaptation before use.

This study has several limitations. First, it was a single-center retrospective analysis. The case mix, nursing-assessment process, accompanying-caregiver system, communication patterns, medical social work resources, and medical insurance policies are institution-specific and may affect model performance and the transportability of both the model and its cutoff. Second, PHQ-9, GAD-7, and the primary outcome were assessed at similar times, so conceptual overlap between predictors and outcome may remain. The sensitivity analysis excluding both scales reduced this concern but could not completely eliminate dependence arising from concurrent measurement. Third, family support, cooperation in physician-patient communication, and perceived economic burden were classified from nursing records. Despite good inter-rater agreement, residual information bias and limited classification validity remain possible. Fourth, single median or mode imputation for variables with <5% missingness may underestimate parameter uncertainty. Fifth, random splitting and bootstrap resampling are both forms of internal validation, and no independent external cohort was used. The 0.586 cutoff, calibration, and feasibility of the clinical workflow require prospective external validation across regions, hospital levels, and nursing-resource settings.

## 5 Conclusions

Unmet psychological care needs were common early in hospitalization among patients with HCC. The risk-stratification model based on the Neuman Systems Model included pain, sleep disturbance, depressive symptoms, anxiety symptoms, family support, cooperation in physician-patient communication, and perceived economic burden. Internal validation showed acceptable discrimination, calibration, and decision-analytic net benefit. The cutoff of 0.586 was internally derived in this cohort and may assist symptom management, psychological screening, family meetings, communication optimization, social-work referral, and dynamic reassessment, but it should not be used as an independent basis for clinical decisions without external validation. The model requires further evaluation in multicenter external cohorts and prospective nursing workflows.

## Data Availability

The original contributions presented in the study are included in the article/**Supplementary Material**. Further inquiries can be directed to the corresponding author.
