# Supplementary material for: Factors associated with unmet psychological care needs and development of a Neuman systems model-based risk prediction model in patients with hepatocellular carcinoma
Source: Front Psychiatry. 2026 Jul 10;17:1887016. doi: 10.3389/fpsyt.2026.1887016 (PMC13396167; doi:10.3389/fpsyt.2026.1887016)
Supplement: Supplementary file 1 [file Supplementaryfile1.docx]

# Supplementary Materials

**Supplementary Table 1. Missing-Data Patterns and Handling Methods**

| **Variable** | **No. missing** | **Missingness (%)** | **Missing-data pattern** | **Handling method** |
| --- | --- | --- | --- | --- |
| Alpha-fetoprotein | 17 | 4.9 | Sporadic | Median imputation |
| Pain NRS score | 5 | 1.4 | Sporadic | Median imputation |
| PHQ-9 score | 7 | 2.0 | Sporadic | Median imputation |
| GAD-7 score | 6 | 1.7 | Sporadic | Median imputation |
| Monthly household income | 27 | 7.8 | Sporadic | Multiple imputation |
| Perceived economic burden | 12 | 3.5 | Sporadic | Mode imputation |
| Convenience of transportation to and from the hospital | 22 | 6.4 | Sporadic | Multiple imputation |
| Family support level | 20 | 5.8 | Sporadic | Multiple imputation |
| Cooperation in physician-patient communication | 18 | 5.2 | Sporadic | Multiple imputation |
| Employment status | 4 | 1.2 | Sporadic | Mode imputation |

**Note:** Continuous variables with <5% missingness were imputed using the median, and categorical variables with <5% missingness were imputed using the mode. Variables with 5%–20% missingness underwent multiple imputation by chained equations. Single median or mode imputation for variables with <5% missingness may underestimate variance; this uncertainty is addressed in the limitations section. NRS, numeric rating scale; PHQ-9, Patient Health Questionnaire-9; GAD-7, Generalized Anxiety Disorder-7.

**Supplementary Table 2. Inter-Rater Agreement for Subjectively Graded Variables**

| **Variable** | **Agreement result** | **Statistical method** |
| --- | --- | --- |
| Family support level | Exact agreement 86.1%; weighted *κ* = 0.82 (95% CI 0.77–0.87) | Weighted Cohen *κ* |
| Cooperation in physician-patient communication | Exact agreement 84.6%; weighted *κ* = 0.79 (95% CI 0.73–0.85) | Weighted Cohen *κ* |
| Perceived economic burden | Exact agreement 88.4%; weighted *κ* = 0.84 (95% CI 0.79–0.89) | Weighted Cohen *κ* |
| Convenience of transportation to and from the hospital | Exact agreement 85.5%; weighted *κ* = 0.81 (95% CI 0.75–0.86) | Weighted Cohen *κ* |
| Regular accompanying caregiver | Exact agreement 92.8%; *κ* = 0.86 (95% CI 0.79–0.93) | Cohen *κ* |

**Note:** Exact agreement is the proportion of classifications for which the two raters agreed completely. *κ* denotes the kappa statistic, and CI denotes confidence interval. Weighted Cohen *κ* was used for ordinal variables, and unweighted Cohen *κ* for binary variables. The 95% CI is the 95% confidence interval for the corresponding *κ* value.

**Supplementary Table 3. Linearity of Continuous Variables With the Logit**

| **Variable** | **Test method** | **Interaction-term *P* value** | **Conclusion** |
| --- | --- | --- | --- |
| Pain NRS score | Box-Tidwell test (1 added to the variable before modeling) | 0.437 | No evidence of departure from linearity with the logit |
| PHQ-9 score | Box-Tidwell test (1 added to the variable before modeling) | 0.281 | No evidence of departure from linearity with the logit |
| GAD-7 score | Box-Tidwell test (1 added to the variable before modeling) | 0.364 | No evidence of departure from linearity with the logit |

**Note:** The Box-Tidwell test was used to assess the linear relationship between each continuous predictor and the outcome logit. An interaction-term *P* value > 0.05 indicates that no statistically significant evidence of nonlinearity was detected, but does not prove perfect linearity. Because the scores could equal 0, 1 was added to each variable before testing. NRS, numeric rating scale; PHQ-9, Patient Health Questionnaire-9; GAD-7, Generalized Anxiety Disorder-7.

**Supplementary Table 4. Stability of LASSO Variable Selection**

| **Variable** | **LASSO selection frequency** | **Selection method** | **Met the ≥70% threshold** |
| --- | --- | --- | --- |
| PHQ-9 score | 20/20 (100.0%) | 10-fold cross-validated LASSO; lambda.1se; *m* = 20 | Yes |
| Sleep disturbance | 20/20 (100.0%) | 10-fold cross-validated LASSO; lambda.1se; *m* = 20 | Yes |
| GAD-7 score | 19/20 (95.0%) | 10-fold cross-validated LASSO; lambda.1se; *m* = 20 | Yes |
| Pain NRS score | 18/20 (90.0%) | 10-fold cross-validated LASSO; lambda.1se; *m* = 20 | Yes |
| Family support level | 18/20 (90.0%) | 10-fold cross-validated LASSO; lambda.1se; *m* = 20 | Yes |
| Cooperation in physician-patient communication | 17/20 (85.0%) | 10-fold cross-validated LASSO; lambda.1se; *m* = 20 | Yes |
| ECOG PS | 15/20 (75.0%) | 10-fold cross-validated LASSO; lambda.1se; *m* = 20 | Yes |
| Perceived economic burden | 15/20 (75.0%) | 10-fold cross-validated LASSO; lambda.1se; *m* = 20 | Yes |
| Decreased appetite | 12/20 (60.0%) | 10-fold cross-validated LASSO; lambda.1se; *m* = 20 | No |
| BCLC stage | 11/20 (55.0%) | 10-fold cross-validated LASSO; lambda.1se; *m* = 20 | No |
| Fatigue | 10/20 (50.0%) | 10-fold cross-validated LASSO; lambda.1se; *m* = 20 | No |
| Primary treatment plan during the current hospitalization | 9/20 (45.0%) | 10-fold cross-validated LASSO; lambda.1se; *m* = 20 | No |
| Ascites | 8/20 (40.0%) | 10-fold cross-validated LASSO; lambda.1se; *m* = 20 | No |
| Portal vein tumor thrombus | 7/20 (35.0%) | 10-fold cross-validated LASSO; lambda.1se; *m* = 20 | No |
| Monthly household income | 6/20 (30.0%) | 10-fold cross-validated LASSO; lambda.1se; *m* = 20 | No |

Note: LASSO, least absolute shrinkage and selection operator; *m*, number of multiply imputed datasets; PHQ-9, Patient Health Questionnaire-9; GAD-7, Generalized Anxiety Disorder-7; NRS, numeric rating scale; ECOG PS, Eastern Cooperative Oncology Group performance status; BCLC, Barcelona Clinic Liver Cancer. Ten-fold cross-validation was used to select the penalty parameter. The 1-standard-error criterion (lambda.1se) identifies the largest penalty parameter for which the cross-validation error is within 1 standard error of the minimum. The prespecified selection-frequency threshold was ≥70%. Selection frequencies for categorical variables with more than two levels were calculated at the overall-variable level. Frequencies indicate the number and proportion of the 20 imputed datasets in which each variable was selected.

**Supplementary Table 5. Sensitivity Analysis Excluding PHQ-9 and GAD-7**

| **Metric** | **Value** | **Statistical method** | ***P* value** |
| --- | --- | --- | --- |
| Training-set AUC | 0.776 (0.715–0.837) | ROC analysis | — |
| Validation-set AUC | 0.754 (0.660–0.834) | ROC analysis | — |
| Brier score | Training set 0.189; validation set 0.199 | Brier score | — |
| Hosmer-Lemeshow test | Training set 0.571; validation set 0.682 | HL test | 0.571/0.682 |
| Pain NRS score | OR = 1.29 (1.12–1.49) | Multivariable logistic regression | <0.001 |
| Sleep disturbance | OR = 2.32 (1.40–3.86) | Multivariable logistic regression | 0.001 |
| Poor family support | OR = 2.56 (1.34–4.88) | Multivariable logistic regression | 0.004 |
| Poor cooperation in physician-patient communication | OR = 2.73 (1.32–5.63) | Multivariable logistic regression | 0.007 |
| Heavy perceived economic burden | OR = 2.06 (1.15–3.69) | Multivariable logistic regression | 0.015 |

Note: The model was refitted after excluding PHQ-9 and GAD-7. PHQ-9, Patient Health Questionnaire-9; GAD-7, Generalized Anxiety Disorder-7; NRS, numeric rating scale; AUC, area under the receiver operating characteristic curve; ROC, receiver operating characteristic; OR, odds ratio; CI, confidence interval; HL, Hosmer-Lemeshow. The Brier score measures prediction-probability error. All *P* values are two-sided.

**Supplementary Table 6. Sensitivity Analysis Using the Strict Outcome Definition**

| **Metric** | **Value** | **Statistical method** |
| --- | --- | --- |
| Primary outcome definition | Score ≥3 on any item in the psychological domain; 205/345 (59.4%) | SCNS-SF34 binary classification |
| Strict outcome definition | Scores ≥3 on at least two items in the psychological domain or a score ≥4 on any such item; 169/345 (49.0%) | Sensitivity analysis |
| Strict-outcome model AUC | Training set 0.835; validation set 0.811 | Repeated LASSO + logistic regression + ROC analysis |
| Direction of principal predictors | Consistent with the primary model | Multivariable logistic regression |

**Note:** SCNS-SF34, Supportive Care Needs Survey Short Form-34; LASSO, least absolute shrinkage and selection operator; logistic regression was used to model the binary outcome; ROC, receiver operating characteristic; AUC, area under the receiver operating characteristic curve. The strict outcome definition was used to evaluate the robustness of the primary-outcome threshold.
